# A fatal case of cytomegalovirus pneumonia with coexisting *aspergillus* infection in a immunodeficient patient

**DOI:** 10.1093/omcr/omaf060

**Published:** 2025-05-28

**Authors:** Kunlun Huang, Shaosen Chen

**Affiliations:** The Second People’s Hospital of Foshan, Department of Respiratory and Critical Care Medicine, 78 Weiguo Rd, Chancheng, Foshan, 528000 Guangdong, P.R. China; The Second People’s Hospital of Foshan, Department of Respiratory and Critical Care Medicine, 78 Weiguo Rd, Chancheng, Foshan, 528000 Guangdong, P.R. China

**Keywords:** cytomegalovirus, aspergillosis, immunodeficient patients, pneumonia, case report

## Abstract

**Introduction**: Cytomegalovirus (CMV) disease is common among transplant patients, who are also prone to secondary bacterial or fungal infections. However, coinfection in immunodeficient patients is rare and often makes diagnosis and treatment challenging.

**Patient concerns**: The patient was an older woman with low immune function but was not a transplant patient.

**Diagnosis**: The patient presented with complaints of fever and shortness of breath for 1 day. After a medical evaluation, she was diagnosed with CMV infection and fungal pneumonia.

**Interventions**: The patient received ceftriaxone + human immunoglobulin + voriconazole treatment.

**Outcome**: The patient’s condition deteriorated and she eventually died of myocardial infarction.

**Conclusion**: For immunocompromised patients, early recognition of coinfections, along with combination medication, maybe a key factor in improving prognosis.

Infection with cytomegalovirus (CMV) is ubiquitous, and most people will develop a primary infection at some point in their lives [1]. Following the initial infection, the virus becomes latent, usually remaining dormant in the normal host for life. Although it does not cause disease during this period, it persists in the host’s leukocytes (possibly monocytes) and may be transmitted to an uninfected individual [2]. However, when the host is immunosuppressed, the latent virus can be activated, leading to severe CMV disease and secondary infections [3]. Additionally, CMV pneumonia increases the likelihood of simultaneous infections by other pathogens, as the virus can induce significant cellular immunosuppression, creating a conducive environment for bacterial and fungal coinfections [4]. While both CMV and Aspergillus pneumonia have been observed in transplant recipients [5], only a few cases have been recorded in immunodeficient patients. The combined infection is challenging to diagnose and treat, often resulting in a poor prognosis.

## Case report

**Figure 1:**
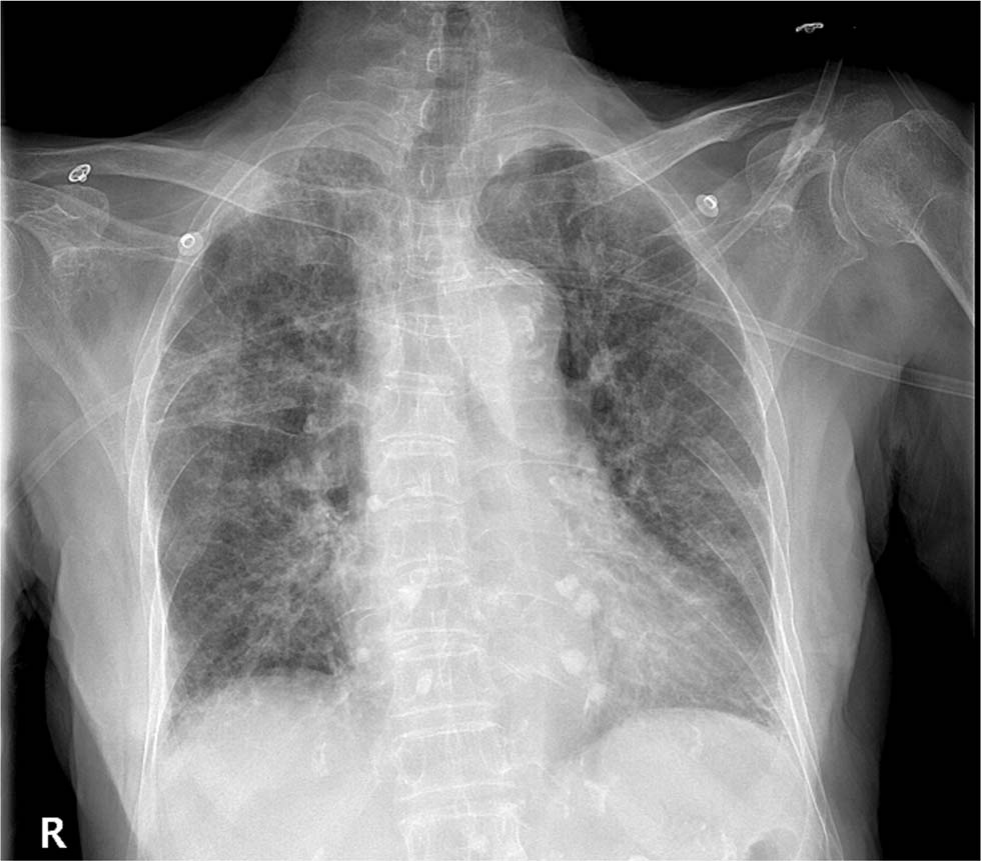
Chest radiograph showed bilateral pneumonia and a few foci of fibrosis in both lower lungs. (57 × 50 mm).

A 93-year-old Chinese woman presented to the clinic with a fever and shortness of breath for 1 day. She was admitted to the hospital for pneumonia treatment based on findings from an outpatient chest X-ray (Fig. 1). Before hospitalization, Prior to hospitalization, the patient was not found to have any chronic diseases, immunocompromised conditions, or COVID-19 infection.

On initial testing, the patient’s white blood cell count (WBC) was 6.95 × 10^9/L, with a monocyte count (MONO) of 0.22 × 10^9/L, neutrophil percentage (NEUT%) of 82.1%,Hemoglobin (Hb) level was 99 g/l, and platelet count (PLT) was 230 × 10^9/L. High-sensitivity C-reactive protein (hs-CRP) was 78.35 mg/l, procalcitonin (PCT) 0.05 ng/ml, interleukin-6 (IL6) 122.66 pg/ml, myoglobin (Myo) 149.0 ng/ml, D-dimer (fluorescence) 726 ng/ml, and erythrocyte sedimentation rate 86 mm/h; These results indicate a mild infection and inflammatory response, with evidence of myocardial damage, while kidney function remains unaffected.

Blood CMV IgG level was 87.20 U/ml, and urine CMV DNA detection < 10^3 IU/ml (polymerase chain reaction (PCR) technology). Human immunodeficiency virus (HIV) antibody/P24 antigen (luminescence method) was 0.10 S/CO. Liver and kidney functions were within normal range. Bronchoalveolar lavage fluid (BALF) assessed by Xpert MTB/RIF (Xpert) was negative, and blood T-spot results were also negative. The patient’s CD4 cell count was 128 cells/mul, the CD8 cell count was 52 cells/mul, and the CD3 cell count was 192 cells/mul. The patient’s CD4, CD8, and CD3 cell counts are significantly below the normal range, suggesting that her immune function is suppressed.

**Figure 2:**
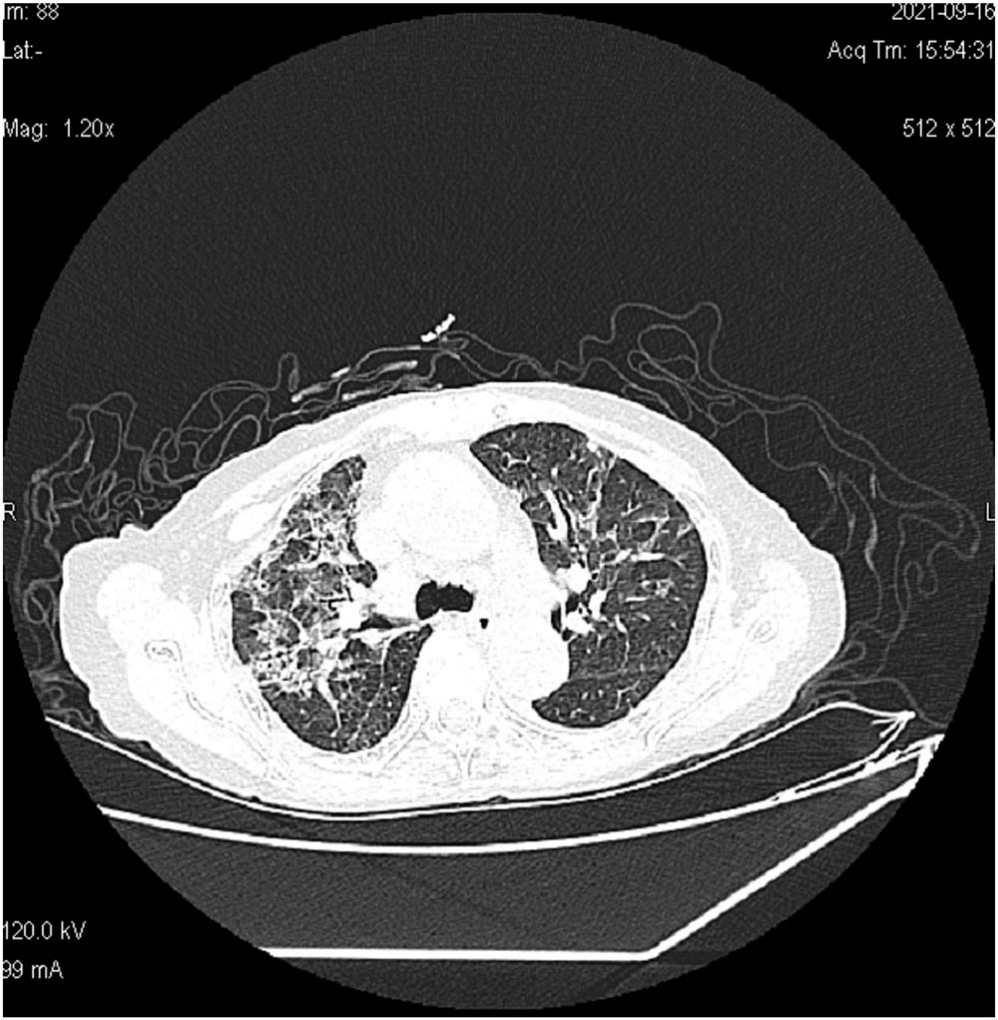
Chest CT showed scattered patches, strips and spots of increased density in both lung, most of the lesions had unclear edges and uneven density. (51 × 52 mm).

The electrocardiogram (ECG) showed sinus rhythm with T-wave abnormalities. The computerized tomography (CT) scan (Fig. 2) revealed a bilateral lung infection, along with slightly dilated bronchi in the middle lobe of the right lung and the upper lobe of the left lung.

### Therapeutic intervention

After the patient’s admission, empirical treatment of ceftriaxone was administered for the infection. On the fifth day of admission, based on the BALF results (metagenomic next-generation sequencing (mNGS): CMV sequences: 5, Relative abundance: 100%), The patient was treated with antiviral therapy, including ganciclovir (2.5 g/q8h) and human immunoglobulin (2.5 g/d). Despite this treatment, the patient experienced recurrent fever, and their shortness of breath worsened on the 10th day of hospitalization.

**Figure 3:**
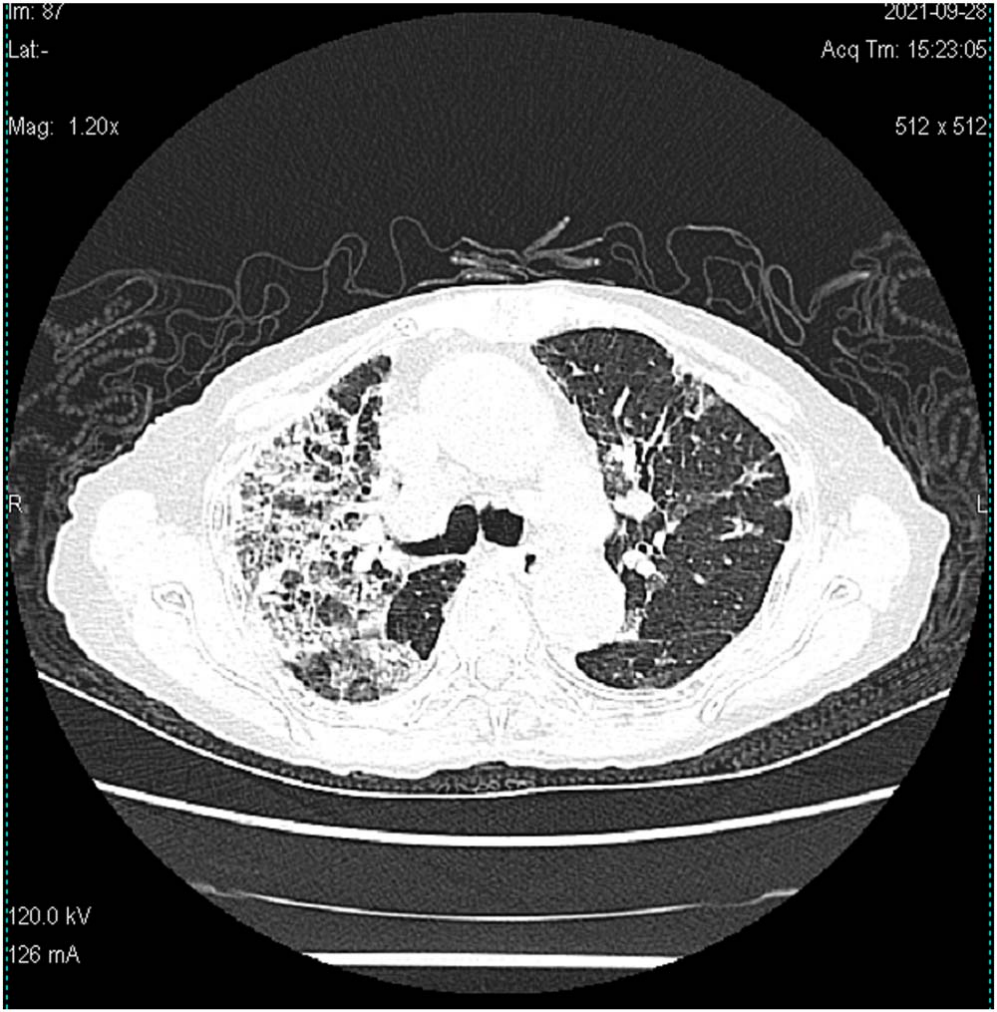
Chest CT showed that bilateral lung lesions increased significantly compared with September 16. (52 × 53 mm).

**Figure 4:**
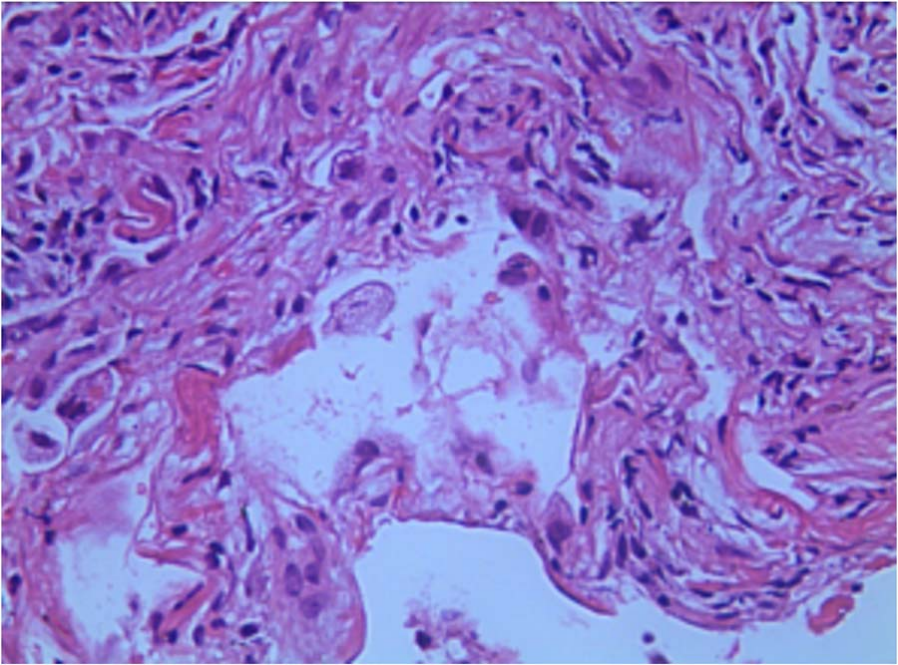
Lung histopathology showed reactive hyperplasia of the alveolar epithelium, with multiple histiocytes and a few chronic inflammatory cells infiltrating in the alveolar space, which were morphologically considered as chronic inflammatory changes (25 × 18 mm).

Subsequent assessments showed PCT <0.04 ng/ml; blood CMV IgM: 0.29 AU/ml; blood CMV IgG: 103.00 U/ml; B-type natriuretic peptide (BNP): 79.2 pg/ml; WBC: 5.71 × 10^9/L; NEUT%: 80.0%; Hb: 87 g/l; PLT: 346 × 10^9/L; hs-CRP: 43.91 mg/l. During this period, ceftriaxone was adjusted to moxifloxacin for anti-bacterial infection treatment owing to urinary infection. On the 13th day, a chest CT (Fig. 3) was performed, revealing a progression of pneumonia. Consequently, diagnostic BAL was performed again under tracheoscopy. BALF was sent for mNGS testing and pathogen culture, and a lung biopsy was performed at the same time. The mNGS test results of BALF showed the presence of *Aspergillus flavus* (number of sequences: 336863, level of confidence: 99%), *Aspergillus fumigatus* (number of sequences: 126752, level of confidence: 99%), CMV (number of sequences: 81, level of confidence: 99%). *Aspergillus* was also cultivated from BALF. Lung histopathology (right upper lung puncture tissue) showed reactive hyperplasia of the alveolar epithelium, with multiple histiocytes and a few chronic inflammatory cells infiltrating the alveolar space, which were morphologically considered as chronic inflammatory changes (Fig. 4). We immediately switched to voriconazole (200 mg q12h, double the first dose) antifungal infection based on the results. No drug-related adverse reactions were observed during treatment.

### Follow-up and outcomes

Despite, administering timely and relevant treatment measures (Table 1) the patient’s condition worsened. Seven days later, the patient experienced a sudden myocardial infarction and died the following day.

**Table 1:** Patient’s summarized timeline.

Dates	Relevant past medical history and interventions	
	Diagnostic testing	Intervention
2021-09-16	bilateral lung infection	‘Ceftriaxone’ anti-infection
2021-09-19	The mNGS test results of BALF showed CMV	Given ‘ganciclovir’ antiviral treatment and ‘human immunoglobulin 2.5 g/d’
2021-10-03	Once again BALF mNGS test results show Aspergillus flavus, *Aspergillus fumigatus*, CMV, TTV-22	“Voriconazole “antifungal infection
2021-10-09	Sudden myocardial infarction	The patient died

## Discussion

The most common manifestations of CMV disease are fever (58%), pneumonia (26.3%), and enterocolitis (15.8%) [6]. The virus can affect the production of various cytokines and chemokines, inhibit natural killer and T-cell responses, and inhibit target humoral immune responses. It may be these immunomodulatory properties that lead to the serious indirect consequences of CMV infection.

CMV infection in individuals with normal immune function does not typically result in immunodeficiency. It is often asymptomatic or causes only mild symptoms, In this case, aside from age, there are no other apparent immunosuppressive factors. We speculate that the patient’s immunodeficiency is a result of immunosenescent, which is the age-related decline in immune function. Patients with CMV pneumonia are particularly prone to fungal pneumonia, especially *Aspergillus* infection. Given that invasive pulmonary aspergillosis progresses rapidly and is associated with high mortality, it becomes crucial to exclude fungal infections in patients with CMV disease. The diagnosis of invasive pulmonary aspergillosis is quite challenging and requires swift action. Early bronchoscopy and histology, culture, and immunohistochemistry of transbronchial biopsy are essential for identifying pulmonary coinfections in immunocompromised patients [5]. In this context, mNGS technology may hold promising prospects in diagnosing such patients [7].

Moreover, studies have shown that patients with invasive pulmonary *Aspergillus* may develop thrombosis problems [8]. Autopsy studies of patients with invasive pulmonary aspergillosis demonstrate vascular damage [9], suggesting a potential link between aspergillosis and thrombosis. Our patient experienced myocardial infarction without any other apparent risk factors except for age. In this case, the patient was found to have normal immune function, no chronic underlying disease, and was considered to be at high risk for CMV infection associated with age. Despite the early diagnosis of CMV infection, and the patient was treated with ‘ganciclovir + human immunoglobulin.’ Additionally, upon detecting the secondary *Aspergillus* infection, ‘voriconazole’ antifungal treatment was promptly initiated. During this treatment, but we used a standardized regimen and did not test the voriconazole concentration because of the restricted nature of the assay; Unfortunately, the treatment’s effectiveness was unsatisfactory, and the patient experienced cardiovascular events in a short period, leading to treatment failure. We believe that the patient’s CMV infection had been long-standing and slowly progressive, contributing to a life-threatening risk factor for secondary *Aspergillus* infection that was insensitive to initial therapy. In such immunocompromised populations, early combined anti-*Aspergillus* infection therapy (triazole combined with echinocandin/polyene combined with echinocandin [10] may improve prognosis. In addition, it is equally important to assess the risk of thrombosis in patients and initiate anticoagulation promptly when necessary.

## Ethical Approval and consent to participate

Not applicable’ for that section.

## Consent to Publication

Consent for publication- written informed consent was obtained from the patients/legal guardian for publication of this case report.

## Gusarantor

Kunlun Huang: As the Guarantor of this paper, I am responsible for the overall integrity of the research. I ensure the accuracy and authenticity of all data and guarantee that the research process adheres to ethical standards. All authors’ contributions have been accurately recorded, and all statements in this paper are true.

## Data Availability

Not applicable’ for that section.
